# Persistence of dysfunctional natural killer cells in adults with high-functioning autism spectrum disorders: stigma/consequence of unresolved early infectious events?

**DOI:** 10.1186/s13229-019-0269-1

**Published:** 2019-05-15

**Authors:** Meriem Bennabi, Nadine Tarantino, Alexandru Gaman, Isabelle Scheid, Rajagopal Krishnamoorthy, Patrice Debré, Arthur Bouleau, Mireille Caralp, Sonia Gueguen, Myriam Ly Le-Moal, Manuel Bouvard, Anouck Amestoy, Richard Delorme, Marion Leboyer, Ryad Tamouza, Vincent Vieillard

**Affiliations:** 1 INSERM, U1160, Hôpital Saint Louis, Paris, France; 2 Fondation FondaMental, Créteil, France; 3 Sorbonne Université, UPMC, INSERM U1135, CNRS ERL8255, Centre d’Immunologie et des Maladies Infectieuses (CIMI-Paris), Paris, France; 4 DHU PePSY, Department of psychiatry, Mondor Hospital, Université Paris Est Créteil, INSERM, U955, Psychiatrie Translationnelle, Créteil, France; 5 Inserm Transfer, Paris, France; 6 French Institute of Health and Medical Research, Paris, France; 7 Institut Roche, Boulogne-Billancourt, France; 8 Institut de Neurosciences Cognitives et Intégratives d’Aquitaine, Université de Bordeaux, Bordeaux, France; 9 DHU Protect, Service de Psychiatrie de l’Enfant et de l’Adolescent, Hôpital Robert Debré, Département de Génétique Humaine et Fonctions Cognitives, Institut Pasteur, Paris, France

**Keywords:** Autism spectrum disorders, High-functioning autism, Natural killer cells, Pathogens

## Abstract

**Background:**

Autism spectrum disorders (ASD) are characterized by abnormal neurodevelopment, genetic, and environmental risk factors, as well as immune dysfunctions. Several lines of evidence suggest alterations in innate immune responses in children with ASD. To address this question in adults with high-functioning ASD (hf-ASD), we sought to investigate the role of natural killer (NK) cells in the persistence of ASD.

**Methods:**

NK cells from 35 adults with hf-ASD were compared to that of 35 healthy controls (HC), selected for the absence of any immune dysfunctions, at different time-points, and over a 2-year follow-up period for four patients. The phenotype and polyfunctional capacities of NK cells were explored according to infectious stigma and clinical parameters (IQ, social, and communication scores).

**Results:**

As compared to HC, NK cells from patients with hf-ASD showed a high level of cell activation (*p* < 0.0001), spontaneous degranulation (*p* < 0.0001), and interferon-gamma production (*p* = 0.0004), whereas they were exhausted after in vitro stimulations (*p* = 0.0006). These data yielded a specific HLA-DR^+^KIR2DL1^+^NKG2C^+^ NK-cell signature. Significant overexpression of NKG2C in hf-ASD patients (*p* = 0.0005), indicative of viral infections, was inversely correlated with the NKp46 receptor level (*r* = − 0.67; *p* < 0.0001), regardless of the IgG status of tested pathogens. Multivariate linear regression analysis also revealed that expression of the late-activating HLA-DR marker was both associated with structural language (*r* = 0.48; *p* = 0.007) and social awareness (*r* = 0.60; *p* = 0.0007) scores in adult patients with hf-ASD, while KIR2DL1 expression correlated with IQ scores (*p* = 0.0083).

**Conclusions:**

This study demonstrates that adults with hf-ASD have specific NK-cell profile. Presence of NKG2C overexpression together with high-level activation of NK cells suggest an association with underlying pathogens, a hypothesis warranting further exploration in future studies.

**Electronic supplementary material:**

The online version of this article (10.1186/s13229-019-0269-1) contains supplementary material, which is available to authorized users.

## Introduction

Autism spectrum disorders (ASD) are heterogeneous neurodevelopmental conditions characterized by deficits in social interactions and repetitive patterns of behavior and interests [1, 2]. The number of reported ASD cases has dramatically increased in recent years, reaching an alarming level of 1 in 68 children in the USA, which represents a 25-fold increase between 1970 and 2012 [3, 4]. Although contributions of several genetic and environmental factors are now well accepted, the etiopathogenesis of ASD remains largely unknown [5]. Immune dysfunction, as reflected by a pro-inflammatory status, is regarded as a significant driver of ASD pathology. Such immune alterations underpinning ASD risk may be driven by maternal infections during the pre-/perinatal period [6–10], possibly interacting with specific immunogenetic backgrounds, such as MET, also called hepatocyte growth factor receptor (HGFR) and human leukocyte antigen (HLA) [11–14]. Together, several alterations in cellular immunity have also been reported in ASD, including a skewed adaptive T cell response toward a T helper 2 phenotype and changes in the cytotoxicity of natural killer (NK) cells [15–17]. Both alterations in immune responses may contribute to an inefficient anti-infectious response, thereby allowing infections to have a significant impact on ASD risk and etiopatho genesis [18]. However, the vast majority of studies have investigated responses in young affected children, with very little information on innate abnormalities, particularly in adults and in high-functioning patients.

NK cell immune subsets constitute a unique niche, given their bridging roles between innate and adaptive immune processes. Upon detection of generic cellular stress signals, transformation, or infection, NK cells acquire immediate effector functions. During infections, NK cells are the critical effectors of innate antiviral immune responses, as demonstrated in cases of inherited NK deficiencies [19]. During immune surveillance, NK cells distinguish their cellular targets from healthy cells via a panel of activating and inhibitory receptors, which recognize ligands specifically induced on “stressed” cells [20]. When activating signals predominate, NK cells produce an array of pro-inflammatory cytokines, such as interferon-gamma (IFN-γ) and tumor necrosis factor-alpha (TNF-α), in parallel to the initiation of their cytotoxic functions [21–23].

Several lines of evidence indicate that NK cells play a role in ASD. Ashwood et al. [24] reported that the absolute count of NK cells was approximately 40% higher in children with low- and high-functioning ASD (hf-ASD), compared to healthy controls. This result is concordant with other studies on children with ASD [15–17]. Interestingly, this increase in NK cells is associated with a deficient response following in vitro stimulation [16], despite the presence of an activating killer-cell immunoglobulin-like receptor (KIR)/HLA complex that could promote immune activation in ASD. Nevertheless, the scarcity of NK-cell studies in adult patients [25] prompted us to evaluate their characteristics in a cohort of adults with hf-ASD.

## Methods and materials

### Participants

Thirty-five adults with hf-ASD, meeting DSM-IV TR or DSM-5 criteria for ASD [26], were systematically recruited under the framework of the French InFoRAutism cohort [27], a bi-centric study. Patients were assessed at inclusion and at two subsequent time points, 12 and 24 months, in two expert centers for ASD under the auspices of the Fondation FondaMental (Créteil and Bordeaux, France) (Table 1). A total IQ score above 70, as assessed with the Wechsler Adult Intelligence Scale (WAIS-III or WAIS-IV), was categorized as high-functioning [28]. Exclusion criteria for patients with hf-ASD were genetic disorders that could generate autism symptomatology (i.e., Angelman syndrome, tuberous sclerosis, fragile X syndrome) and active neurological disorders (e.g., seizure disorders). Adults with ASD were assessed with standardized diagnostic tools, including the French version of the Autism Diagnostic Interview-Revised (ADI-R) and the Autism Diagnosis Observational Schedule (ADOS) [29]. Social and communication scores, core dimensions of ASD symptomatology, were also rated using the Social Responsiveness Scale (SRS) and the Communication Checklist for Adults (CCA), respectively [30, 31]. The SRS scale generates five sub-scores evaluating different aspects of social interaction: social awareness (SAWR), social cognition (SCOG), social communication (SCOM), social motivation (SMOT), and restricted interests and repetitive behaviors (SRRB) (Table 1). The CCA yields three composite scores measuring different aspects of communication, namely language structure, pragmatic skills, and social engagement.

**Table 1:** Demographic and clinical data of patients with hf-ASD

Characteristics	ASD
Number	35
Mean age in years (range)	30 (18–56)
Ratio, male to female	27:8
Mean IQ value (range)	105 (70–146)
Communication Checklist for Adults scale (CCA)	
CCA-LS, (range, mean ± SD)	(2–39), 11 ± 9
CCA-PS, (range, mean ± SD)	(1–50), 18 ± 11
CCA-SE, (range, mean ± SD)	(21–57), 37 ± 9
Social Responsiveness Scale (SRS)	
SAWR, (range, mean ± SD)	(41–92), 60 ± 12
SCOG, (range, mean ± SD)	(41–95), 65 ± 12
SCOM, (range, mean ± SD)	(44–97), 68 ± 11
SMOT, (range, mean ± SD)	(44–91), 68 ± 12
SRRB, (range, mean ± SD)	(45–100), 69 ± 13
SRS T score, (range, mean ± SD)	(45–100), 68 ± 12
Autism Diagnostic Observation Schedule (ADOS)	
Social communication (range, mean ± SD)	(3–21), 11 ± 5
Language and communication (range, mean ± SD)	(1–8), 4 ± 2
Reciprocal social interactions (range, mean ± SD)	(1–14), 7 ± 3

*IQ* intelligence quotient, *CCA-LS* structural language, *CCA-PS* pragmatic behavior, *CCA-SE* social engagement, *SAWR* social awareness, *SCOG* social cognition, *SCOM* social communication, *SMOT* social motivation, *SRRB* restricted interests and repetitive behaviors, *SRS T score* SRS total score

Two consecutive groups of controls were included and tested for their NK-cell signature. We started the study by analyzing control samples recruited under the framework of the InfoR program (InFoR-Ctl). This cohort consisted of 25 individuals selected only based on the absence of personal and familial psychiatric disorders, without any criteria to assess somatic disorders likely related to immune dysfunction, including history of infections and/or presence or history of immune/autoimmune disorders (see Additional file 1: Table S1). As we rapidly observed that NK cells belonging to this cohort presented immune abnormalities, we decided to introduce and use control samples belonging to a pool of over 250 anonymous individuals from the French National Blood Bank (EFS, Paris, France), called EFS-Ctl, currently used as the standard for the routine biological diagnosis of NK lymphoproliferative diseases (i.e., LGL, leukemia, etc.) and for other related immune disorders in the Department of Immunology of the Pitié-Salpêtrière hospital as previously described [32–34]. These blood samples were certified for their absence of common viral infections (i.e., HIV-1 and 2, hepatitis B and C viruses) and lymphocyte dysfunction (absolute value and frequency of subpopulation, activation status) (https://www.legifrance.gouv.fr/eli/arrete/2016/4/5/AFSP1608360A/jo/texte). From these EFS samples, 35 sex- and age-matched EFS-Ctl were randomly selected and served as controls in all experiments.

The study was approved by local Ethical Committees and registered in the public trials registry (NCT02628808). All study participants gave their informed written consent for inclusion in this study.

### HLA and KIR genotyping

HLA and KIR experiments were performed on DNA extracted from total peripheral blood mononuclear cells (PBMCs). HLA genotyping was performed using PCR-sequence-specific oligonucleotide (SSO) Luminex LABTYPE SSO kits designed to recognize all the broad specificities. The Luminex 100 flow analyzer identified HLA alleles via HLA visual 1.0 software, by referring to HLA typing template data for the studied loci, as provided by the manufacturer (OneLambda, Inc., CA). KIR genotyping was performed by PCR, using a combination of specific oligonucleotide primers and internal controls, as previously described [35].

### Flow cytometry analyses

NK-cell subsets were analyzed by flow cytometry within the CD45^+^ lymphocyte population (anti-CD45-Chrome Orange; #J33), gated on the CD3^−^ (anti-CD3-ECD; #UCHT1) and CD56^+^ (anti-CD56-PC7; #N901) population, with an appropriate cocktail of 11 antibodies, including anti-CD159a/NKG2A-APC (#Z199); anti-CD335/NKp46-APC (#BAB281); anti-NKG2D-APC; (#ON72); anti-HLA-DR-PE (#Immu357); anti-CD57-FITC (#S-HCL-1) from Coulter, DNAM-1-FITC (#DX11) from Becton Dickinson, anti-CD337/NKp30-PE (#AF29-4D12), and anti-KIR2DL2/KIR2DL3-APC (#DX27) from Miltenyi Biotech; anti-NKG2C-PE (#134591); anti-KIR2DL1-FITC (#143211); and anti-KIR3DL1-APC (#DX9) from R&D systems.

At least 10,000 CD45^+^CD3^−^CD56^+^ cells were analyzed on a Gallios cytometer (Beckman Coulter). Expression of each marker was measured as a percentage of the total CD3^−^CD56^+^ NK cells, as described earlier [36]. A hierarchical clustering was applied for the 11 NK cell-specific phenotypic markers, with the results displayed using the GENESIS program (software available at www.genome.tugraz.at), as described previously [37, 38].

### Serology of cytomegalovirus and other pathogens

Cytomegalovirus (CMV) immunoglobulin (Ig)G serology was analyzed for the 35 hf-ASD subjects, using a Luminex-based technology (Bioplex 2200 ToRC IgG Reagent Pack), according to the manufacturer’s instructions. A confirmatory assessment of the CMV status, together with the serological screening for 23 other common pathogens, was performed for ASD subjects and EFS-Ctl controls using commercially available ELISA kits (see Additional file 1: methods).

## Degranulation and intracellular production of cytokines

NK cell degranulation was investigated using assays that were able to detect the CD107a marker, on either overnight-cultured PBMCs in the presence of IL-12 (10 ng/ml) plus IL-18 (100 ng/ml) or on PBMCs incubated with K562 target cells, at an effector to target (E:T) cell ratio of 1:1 [32]. Cells were incubated in the presence of an anti-CD107a monoclonal antibody (mAb) (FITC; #H4A3; Becton Dickinson) for 1 h, followed by a 5-h incubation after the addition of Golgi Stop and Golgi Plug solutions (BD Biosciences). For the analysis of intracellular cytokine production, cells were stained with cell-surface markers (anti-CD3 and anti-CD56 mAbs), fixed, permeabilized with a cytofix/cytoperm kit (Becton Dickinson), and then stained with mAbs for anti-IFN-γ (Alexa Fluor 700; #B27; Becton Dickinson) and anti-TNF-α mAbs (eFluor450; #Mab11; eBiosciences), as previously described [34, 39].

### Statistical analysis

Statistical analyses were performed with Prism-5 software (GraphPad Software). The nonparametric Mann-Whitney tests were performed for individual comparisons of unrelated samples from hf-ASD patients and EFS-Ctl. Nonparametric correlations were assessed by the determination of the Spearman’s rank correlation coefficient. Linear regressions models were used to explore associations between NKG2A; KIR2DL1; NKG2C; HLA-DR; NKp46; and CD107a with age, sex, CCA, SRS, and IQ. Analyses were conducted using Stata 13.

## Results

### NK-cell signature in hf-ASD patients

**Fig. 1:**
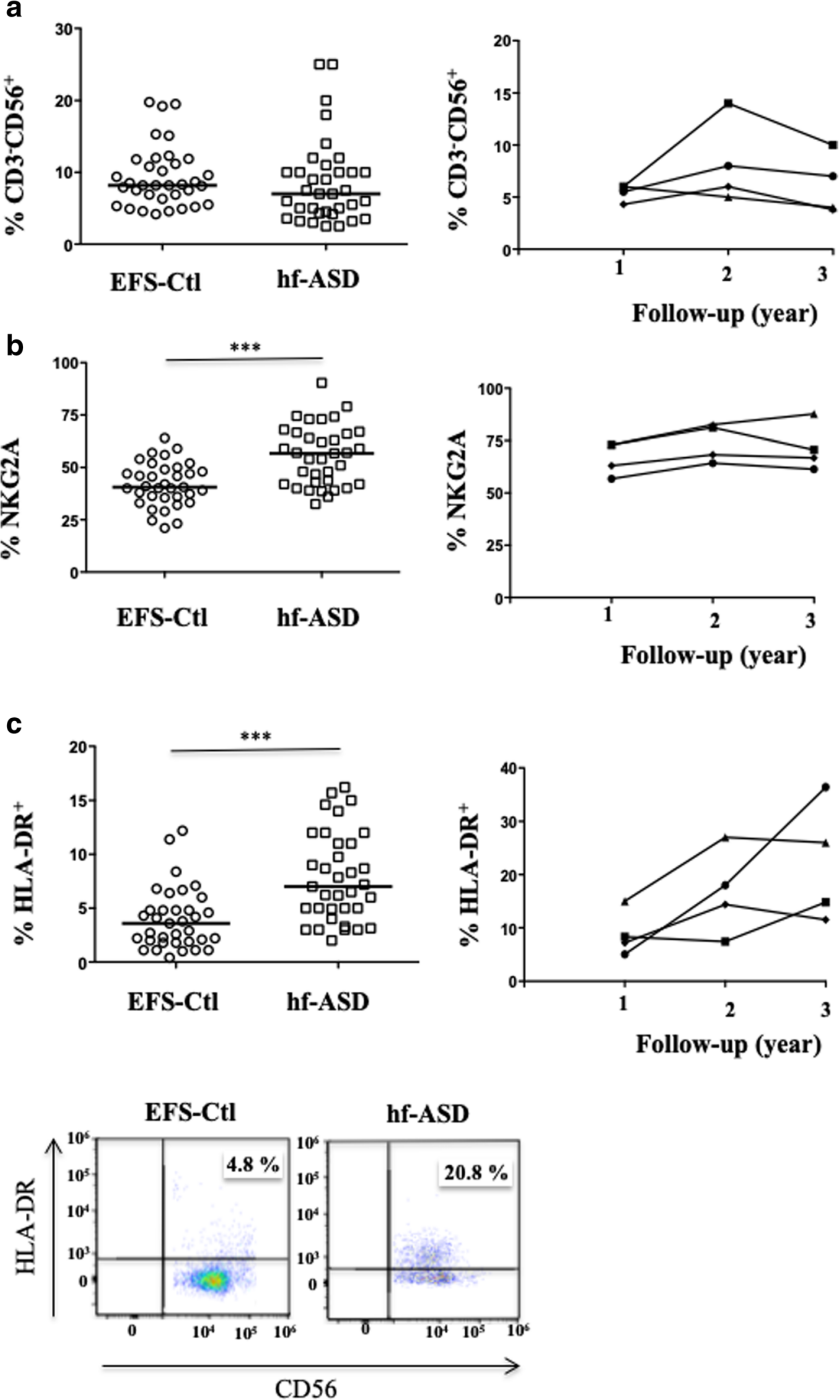
Extensive phenotypic analysis of NK cells in patients with hf-ASD. **a** The frequency of CD3^−^CD56^+^ cells within the CD45^+^ lymphocyte gate in 35 patients with hf-ASD and 35 age- and sex-matched healthy controls from the French National Blood Service (EFS-Ctl), with a follow-up over a 3-year period in four hf-ASD patients. **b** The frequency of the immature marker, NKG2A, within the CD3^−^CD56^+^ NK cells gate in 35 patients with hf-ASD and 35 EFS-Ctl, *p < 0.05 (Mann-Whitney test), and the follow-up during a 3-year period in four hf-ASD patients. **c** The frequency of the late activation marker HLA-DR in 35 patients with hf-ASD and 33 EFS-Ctl, ***p < 0.0001 (Mann-Whitney test). Representative samples are shown in the right panels

The present study was conducted on 35 adults with hf-ASD and 35 age- and sex-matched healthy controls, EFS-Ctl (Table 1). The distribution of CD3^−^CD56^+^ NK cells, as measured by flow cytometry, was comparable in hf-ASD patients and EFS-Ctl controls at inclusion and during the follow-up period (Fig. 1a), which agreed with data from previous studies [16, 17]. In addition, the analysis of the CD56^dim^ and the CD56^bright^ subsets showed a normal distribution of the two subsets, except for a few CD56^bright^ (see Additional file 1: Figure S1a). However, the frequency of NK cells expressing the immature marker, NKG2A, was significantly increased in patients with hf-ASD (*p* = 0.01), as compared to EFS-Ctl at inclusion, and it remained elevated over the 2-year follow-up period (Fig. 1b).

Importantly, the frequency of NK cells expressing the late cell-activation marker, HLA-DR, was significantly increased in patients with hf-ASD compared to the EFS-Ctl control group (*p* < 0.0001) (Fig. 1c). In the four tested hf-ASD patients that were observed over the follow-up period, the high expression level was stable, or slightly increased, both in CD56^dim^ and CD56^bright^ NK cell compartments (see Additional file 1: Figure S1b), but not in the CD3^+^ T and CD19^+^ B cell subsets (data not shown), indicating that lymphocyte activation was exclusively restricted to the NK cells.

**Fig. 2:**
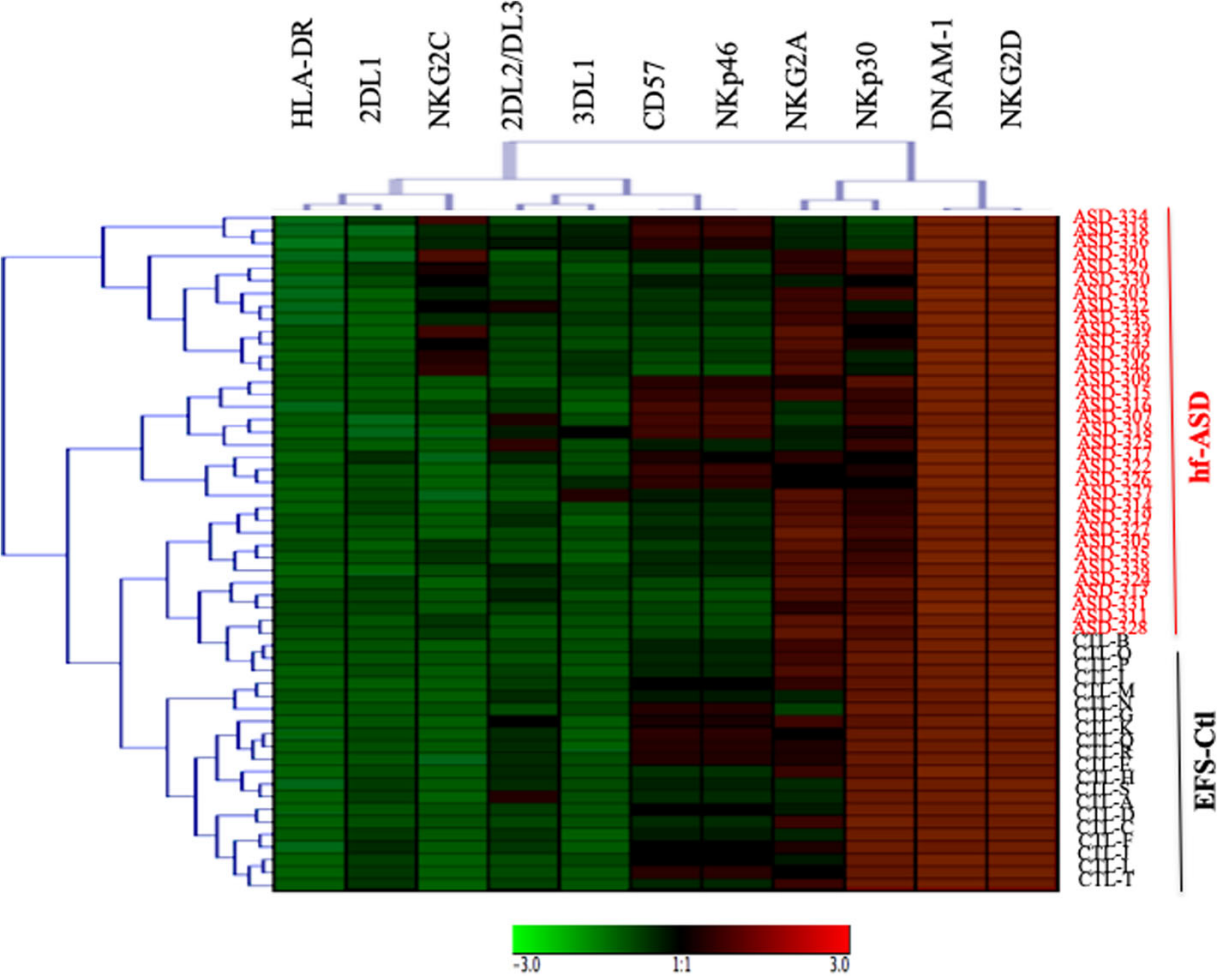
Hierarchical clustering analysis of 11 NK-cell markers in 34 patients with ASD and 20 age- and sex-matched healthy controls from the French National Blood Service (EFS-Ctl). Each column is dedicated to a distinct NK marker. The color of each square reflects the percentage of expression of the corresponding marker in each individual. The values measured for all samples were color-displayed and rank-ordered considering the healthy donors’ median as a reference: green indicates inferior to median, and red indicates superior to median with values that ranged between − 3 and + 3. The analysis was performed with the GENESIS program (available at http://www.genome.turgaz.at)

**Fig. 3:**
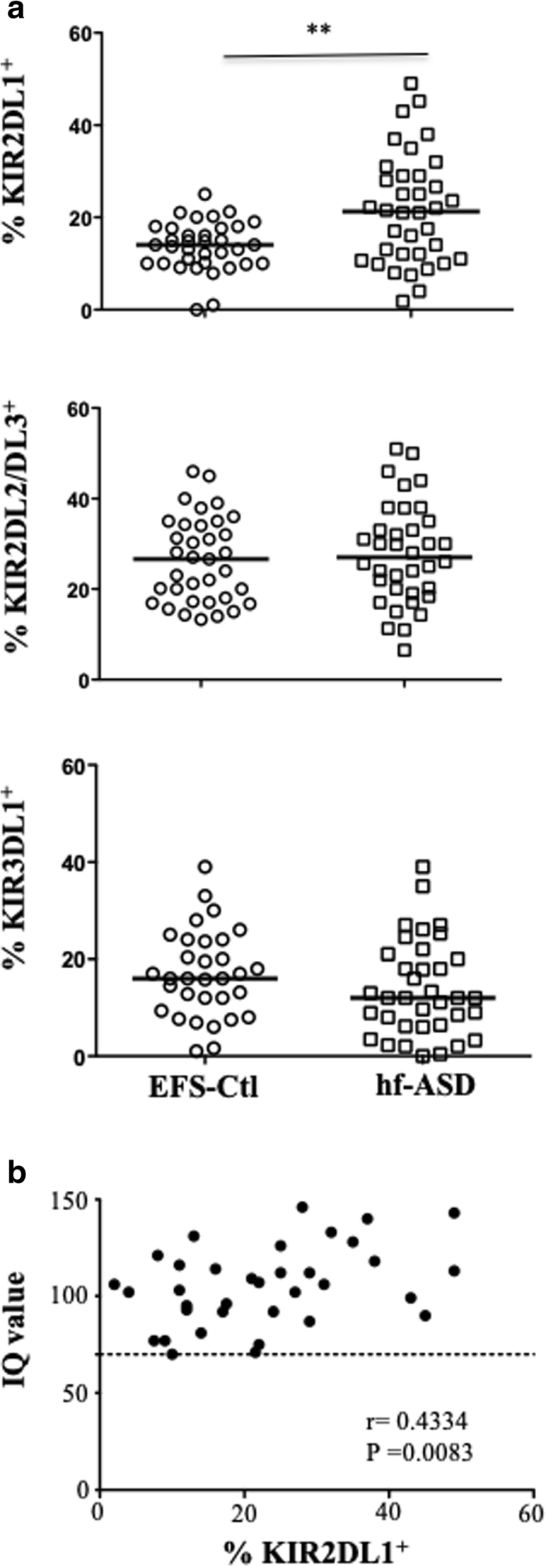
The association of KIR2DL1 expression and intelligence quotient (IQ) score in patients with hf-ASD. **a** The frequency of KIR2DL1^+^, KIR2DL2/2DL3^+^, and KIR3DL1^+^ cells gated on CD3^−^CD56^+^ NK cells from 35 patients with ASD and 35 age- and sex-matched healthy controls from the French National Blood Service (EFS-Ctl), *p* < 0.05 (Mann-Whitney test). **b** The correlation between KIR2DL1^+^ NK cells in 35 ASD patients and IQ score. The dotted line indicates that IQ values were equal to 70

To further investigate the NK cell-activation status, a large-scale flow-cytometric analysis of the frequency of 11 NK-cell markers was first carried out (Fig. 2). A hierarchical clustering analysis of the data revealed that the expression pattern of NK cells in hf-ASD patients was distinct from that observed in EFS-Ctl (Fig. 2), suggesting a specific hf-ASD NK-cell phenotype. This analysis also revealed a specific clustering of the HLA-DR, KIR2DL1, and NKG2C markers (Fig. 2). More precisely, when hf-ASD was compared to EFS-Ctl, KIR2DL1 was significantly increased (*p* = 0.005), while KIR2DL2/DL3 and KIR3DL1 were unchanged (Fig. 3a). Of note, and in agreement with previous studies [14], some activating KIR genes (*KIR2DS2* and *KIR2DS3*) were observed more in hf-ASD patients than in EFS-Ctl (see Additional file 1: Table S2). More importantly, the modulation of inhibitory KIR receptors and their ligands (HLA-C1, C2, or Bw4) was not found predictable by any KIR or HLA genetic profile, both in hf-ASD and EFS-Ctl (see Additional file 1: Table S2).

**Fig. 4:**
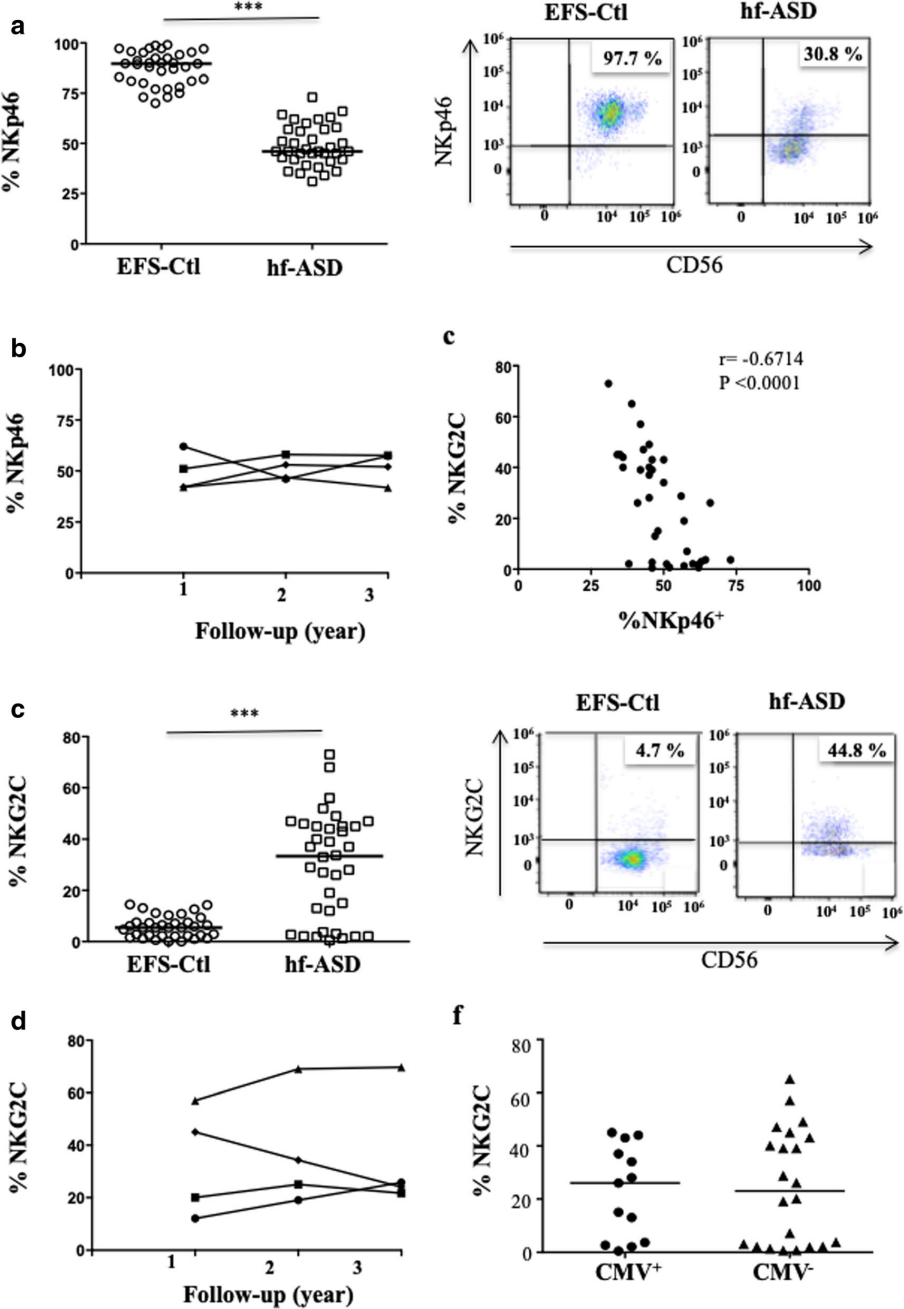
The atypical expansion of NKG2C^+^ NK cells in patients with hf-ASD. **a** The frequency of NKp46^+^ cells gated on CD3^−^CD56^+^ NK cells in 35 ASD patients and 35 age- and sex-matched healthy controls from the French National Blood Service (EFS-Ctl), ***p < 0.0001(Mann-Whitney test). Representative samples in right panels. **b** The follow-up of NKp46 frequency on CD3^−^CD56^+^ NK cells over a 2-year period in four hf-ASD patients. **c** The correlation between NKG2C and NKp46 in NK cells in 35 hf-ASD patients. **d** The frequency of NKG2C^+^ cells gated on CD3^−^CD56^+^ NK cells in 35 hf-ASD patients and 35 EFS-Ctl, *p < 0.05 (Mann-Whitney test). Representative samples are shown the in right panels. **e** The follow-up of NKG2C frequency on CD3^−^CD56^+^ NK cells over a 2-year period in four hf-ASD patients. **f** NKG2C expression on NK cells in CMV seronegative (*n* = 22) and seropositive patients (*n* = 13)

We next investigated an extended panel of activating NK receptors. Most of these NK cell-activating receptors, such as CD57, NKp30, NKG2D, and DNAM-1, were similarly expressed in NK cells from hf-ASD patients and the EFS-Ctl group (see Additional file 1: Figure S2). In contrast, Fig. 4a shows that NKp46 exhibit lower expression levels in hf-ASD patients compared to EFS-Ctl (*p* < 0.0001; Fig. 4a), during the follow-up period (Fig. 4b), and more specifically in the CD56^dim^ NK-cell subset (see Additional file 1: Figure S1c). However, NKp46 expression was inversely correlated with that of NKG2C, another activating NK receptor (*r* = − 0.6714; *p* < 0.0001; Fig. 4c). Interestingly, we observed that NKG2C expression was significantly increased in 24 of the 35 hf-ASD participants, as compared to EFS-Ctl (19–68% vs 0.0–14.5%; *p* < 0.0001 in ASD and in EFS-Ctl, respectively) (Fig. 4d). This increased NKG2C expression level remained stable over the follow-up period (Fig. 4e), which is primarily typical of CMV seropositive individuals and to a lesser extent for other viral infections, including HIV-1, hantavirus, chikungunya virus, and viral hepatitis [33, 36]. Strikingly, NKG2C expression in hf-ASD patients was found to be similar in both CMV seropositive and in seronegative cases (Fig. 4f), likely excluding a CMV-mediated process. Consequently, serum IgG antibodies directed against 23 other common pathogens were screened, with the a priori expectation that no association would be demonstrated. Indeed, only a trend toward an association between the presence of IgG anti-hepatitis A virus, anti-herpes simplex virus (HSV) type 2, and anti-Brucella were observed (see Additional file 1: Table S3). In addition, none of these common pathogens showed a significant correlation with NKG2C expression on NK cells. Overall, the results surprisingly indicate that adults with hf-ASD have an elevated rate of highly activated and dysfunctional NK cells, directly or indirectly triggered by a pathogen, that remains to be determined or merely by another yet to be identified mechanism.

### NK-cell functions in hf-ASD patients

**Fig. 5:**
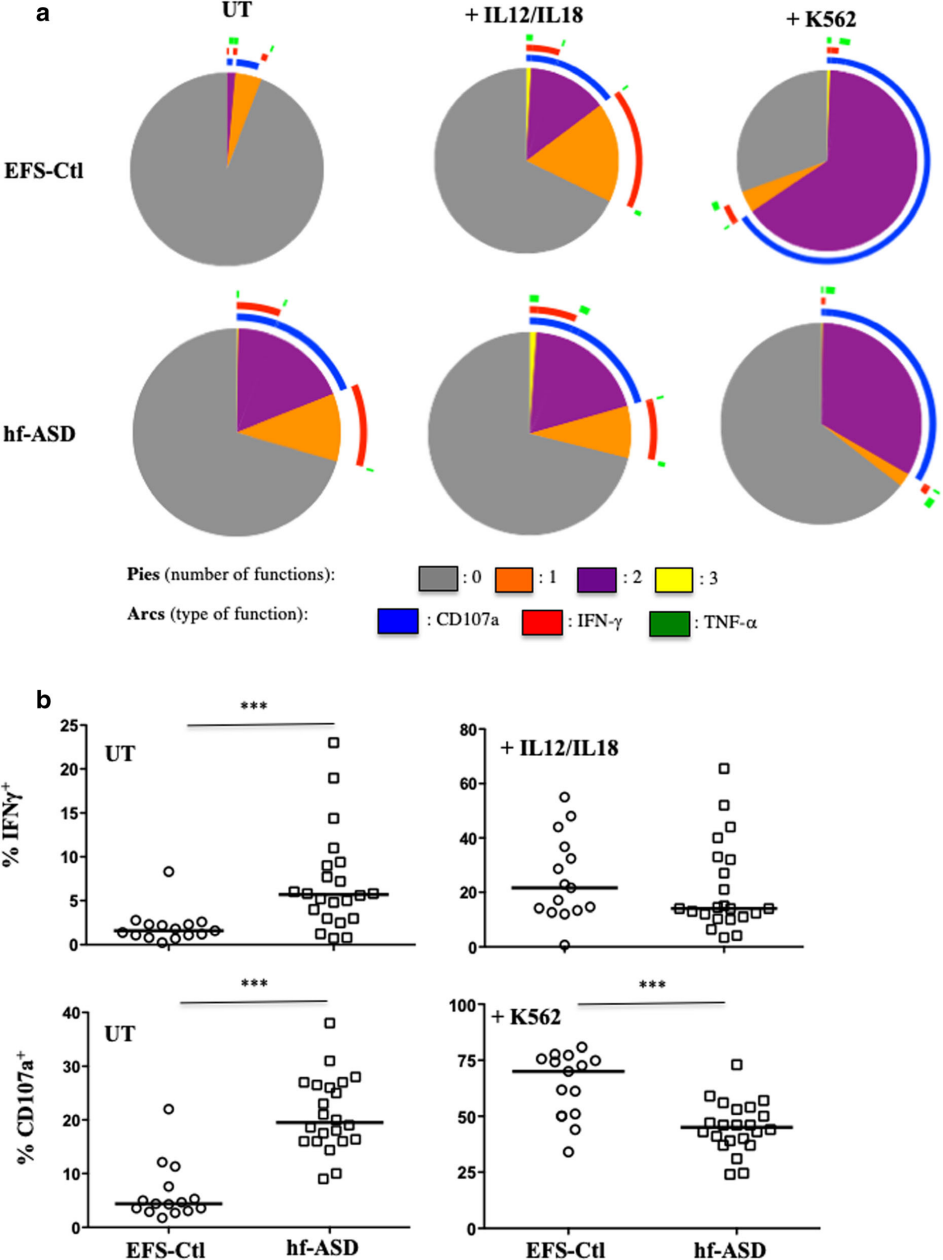
Polyfunctional activity of NK cells from hf-ASD patients. **a** Polyfunctionality of CD3^−^CD56^+^ NK cells (degranulation and production of IFN-γ and/or TNF-α), 20 patients with hf-ASD (median), compared to 15 age- and sex-matched healthy controls from the French National Blood Service (EFS-Ctl, median) at entry. Assays were performed in cells: untreated (UT), IL-12 + IL-18 overnight stimulation, or in the presence of K562 target cells. The values were analyzed with a Boolean gate algorithm (FlowJo; Tree Star, Ashland, OR, USA). Pie and arc charts were generated using SPICE software (National Institute of Allergy and Infectious Diseases freeware). Pies represent the frequency of NK cells positive for 0, 1, 2, or 3 responses (to CD107a, IFN-γ, and TNF-α). Arcs depict cellular functions as functional or polyfunctional. **b** Intracellular of production of IFN-γ among UT or IL-12 + IL-18 overnight stimulation, and degranulation of NK cells measured by cell-surface expression of CD107a in CD3^−^CD56^+^ NK cells from 22 hf-ASD patients and 15 EFS-Ctl, tested in absence (UT) or in the presence of the standard K562 target cells (ratio 1:1), ***p **<** 0.0001 (Mann-Whitney test)

To better understand the possible mechanisms associated with these abnormal immune phenotypes, polyfunctional assays were performed to test the ability of NK cells to simultaneously degranulate and produce cytokines (IFN-γ and/or TNF-α). These assays were carried out in untreated NK cells, in IL-12/IL-18-treated NK cells, and in NK cells after stimulation with K562 target cells. Unexpectedly, the polyfunctional activity of NK cells from hf-ASD patients compared to EFS-Ctl was higher without any treatment (Fig. 5a), including a significant increase in intracellular IFN-γ production and a more pronounced degranulation process (Fig. 5b; *p* < 0.0001). Of note, TNF-α expression was almost undetectable in unstimulated cells of both hf-ASD patients and EFS-Ctl subjects (see Additional file 1: Figure S3a, S3b). It is important to highlight that after IL12/IL18 treatment or stimulation with K562 target cells, as seen in previous studies [16, 17], NK cells of hf-ASD patients showed a decrease in IFN-γ production and a drastic reduction in degranulation levels compared to EFS-Ctl NK cells, as reflected by the very low levels of the CD107a marker (Fig. 5a, b; *p* = 0.0008 for both). Similar profiles of cytokine production and degranulation were observed over the 2-year follow-up period (see Additional file 1: Figure S3c).

Overall, the data indicates an exhaustion status of NK-cell functional responsiveness in patients with hf-ASD, likely associated with a non-stimulated chronic cell-activation state.

### Relationship between NK cells and IQ, communication and social scores of hf-ASD

Only HLA-DR and NKp46 markers were associated with social communication (CCA) and social reciprocity (SRS) scores. A significant correlation was found between NK-HLA-DR expression and both CCA-LS (structural language) (*r* = 0.48; *p* = 0.007) and SAWR (social awareness) (*r* = 0.60; *p* = 0.0007) scores, while results for NKp46 only approached significance, following multivariate analysis (Table 2). Interestingly, the analysis of potential relationships between IQ scores and NK-cell characteristics showed a statistically significant association with KIR2DL1 (Spearman test; *r* = 0.4334; *p* = 0.008) (Fig. 3b), a finding further confirmed by linear multivariate analysis, adjusting for age and gender (95% CI [0.1140; 0.5272]; *p* = 0.003).

**Table 2:** Phenotypic and clinical correlations in hf-ASD patients

	Communication Checklist for Adult scale (CCA)			Social Responsiveness Scale (SRS)					
Markers	CCA-LS	CCA-PS	CCA-SE	SAWR	SCOG	SCOM	SMOT	SRRB	T score
NKp46	ns	ns	*p* = 0.03 *r* = 0.4	*p* = 0.04 *r* = 0.38	*p* = 0.02 *r* = −0.4	ns	ns	ns	*p* = 0.04 *r* = − 0.36
HLA-DR	*p* = 0.007 *r* = 0.48	ns	ns	*p* = 0.0007 *r* = 0.6	ns	*p* = 0.01 *r* = 0.44	ns	ns	ns

*CCA-LS* structural language, *CCA-PS* pragmatic behavior, *CCA-SE* social engagement, *SAWR* social awareness, *SCOG* social cognition, *SCOM* social communication, *SMOT* social motivation, *SRRB* restricted interests and repetitive behaviors, *SRS T score* SRS total score, *ns* non-significant

## Discussion

The present study provides novel insights in the exploration of immune dysfunction in ASD by implicating NK cells in the pathophysiology of adult patients with hf-ASD. In contrast to previously reported data in cohorts of children with ASD [15–17, 24], we observed that the frequency of NK cells in adult patients with hf-ASD tends to remain similar to that of healthy controls. This discrepancy could be related to the age of patients. Indeed, two large-scale studies have quantified the distribution of lymphocyte subsets in the peripheral blood of healthy children and showed that both total NK cell numbers and percentages differ from birth through late adolescence [40, 41]. This variation may be also indicative of the already reported phenotypic differences in NK cells between children and adults [42]. Altogether, these results highlight the importance of considering socio-demographic characteristics of the studied sample before any comparison with other data.

Interestingly, in a substantial proportion of hf-ASD patients (61% of cases), we found NK cells characterized by the persistence of an uncommon CD56^dim^HLA-DR^+^NKG2C^high^KIR2DL1^+^ phenotype signature. This signature was found to be associated with core ASD clinical dimensions and remained stable during the 2-year follow-up period in the tested samples. These data are highly evocative of a viral pathogenic involvement in ASD risk, since following pathogen exposure, the NK cell repertoire is known to display several distinct characteristics, including expansion of cells expressing the activating NKG2C receptor, a self-specific inhibitory KIR receptor (KIR2DL1), and low expression levels of NKp46 [43]. The expansion of NKG2C^+^ NK cells is most commonly evident in CMV seropositive individuals [43, 44]. In contrast, we observed that the expansion of NKG2C^+^ NK cells occurred irrespective of their CMV status in our sample of patients with hf-ASD. Although the differentiation and proliferation of NKG2C^+^ NK cells in ASD requires further investigation, the present results may indicate a yet to be identified ASD-specific pathogen driving NKG2C^+^ receptor overexpression. As several studies implicate pre-, peri-, and postnatal infections for ASD risk [6–9, 45], the present data gives some support to non-resolved infectious events in ASD, possibly arising from suboptimal, genetically-determined, anti-infectious responses, and/or from other ASD-related processes. A permanent state of NK cell activation and inflammation, possibly having deleterious central nervous system (CNS) consequences, may arise from this hyper-activation.

The other observation that deserves to be highlighted pertains to the abnormal ability of adult hf-ASD NK cells to degranulate and to produce abnormal amounts of IFN-γ at a steady state level, while becoming hypo-functional/exhausted when challenged by treatment with IL-12/IL-18 or stimulation with K562 target cells. Such characteristics have been shown to occur during childhood in patients with ASD [15–17, 24], as well as in a range of other psychiatric disorders, including obsessive-compulsive disorder, chronic stress, and depression [46, 47], all reflecting alterations in systemic NK cell activity. The presence of NK cells in the CNS may suggest more direct central effects of their dysfunctions, whose exploration will require further investigation [48].

The state of NK cell exhaustion in adults with hf-ASD may be due to heightened levels of peripheral NK cell inflammation/activation, in accordance with the “discontinuity theory” [49], in which the immune system responds adequately to sudden changes in antigenic stimulation but becomes tolerant after slow or continuous stimulations. This could also merely reflect abnormal ontogenetic development of the CD56^bright^ and CD56^dim^ subsets of NK cells, given their respective capacity of cytokine production and cytotoxicity, with implication in ASD development. This is likely reflected by the trends observed on associations between elevated levels of activated HLA-DR^+^ NK cells and core phenotypic dimensions of hf-ASD, especially structural language (CCA-LS) and social awareness (SAWR), which implicates alterations in NK-cell functioning in ASD. Such data may also fit well with wider observations, including: (1) previous psychobiological models linking inflammation, lower social status, poor physical status, and emotional distress [50]; (2) ligands of the activating NKG2D NK-cell receptor are expressed in neuronal stem cells; and (3) NK cells are essential for normal brain development. Hence, any functional impairment of NK cells during critical neurodevelopmental windows may affect neurogenesis, resulting in adverse consequences for brain development [51, 52].

Finally, according to the fine-tuned equilibrium between inhibitory and activating NK cell receptors, the observed high expression of the inhibitory KIR2DL1 receptor (for which HLA-C2 is a natural ligand) and its relationship with IQ, fits to the known relationship between ASD and HLA alleles [53, 54], as well as to the genetic association between major histocompatibility complex single nucleotide polymorphisms and IQ scores in ASD [52]. Other studies showed that the frequency of several activating KIR genes and their cognate HLA ligands is higher in ASD children and their mothers [55, 56]. Furthermore, the low-expressor genotype of the immuno-modulatory non-classical HLA-G was consistently found to be more prevalent in ASD patients belonging to different population groups [57–59]. Altogether, the finding of this study reinforces the abovementioned genetic link established with the commonly observed immune activation status in ASD. Given that, it will be of interest in future studies to investigate the presence of such correlations in cohorts of adult and children with hf- and non-hf-ASD.

Overall, the permanent activation of NK cells in ASD may contribute to a vicious cycle of persistent degranulation potential, heightened inflammation, and neuro-immune damage and dysfunction.

## Limitations

This study stressed the importance of a fine-tuned design for the inclusion criteria of controls in the immune analysis of psychiatric disorders. Indeed, in psychiatric studies, controls are usually included according to the absence of psychiatric conditions using only after psychiatric assessments without any biological and/or somatic screening. By contrast, in immunological settings, controls are included based on the strict absence of any abnormality concerning either biological markers of immune dysfunction(s) or the existence of any past and present immune disorders but without any assessment of psychiatric disorders. In France, according to the international requirements, immunological analysis is always based on a comparison of patients’ samples with certified samples of healthy donors from blood banks, i.e., EFS. Our observations reveal, for the first time, a dysfunction of the NK cell compartment in a subgroup of patients with ASD. It will thus be important to replicate these results in future studies with new cohorts of patients, matched with healthy donors, screened both for neuropsychiatric and immune disorders.

## Conclusions

The data presented here demonstrates that a chronic NK cell inflammatory/activation process in adult hf-ASD is associated with core autism symptomatology. The early developmental etiopathogenesis that drives these long-lasting changes, as well as a better understanding of such pathophysiological processes that occur later in life in patients with ASD, may help in the development of new targets for therapeutic interventions.

## Additional file

Additional file 1: **Table S1.** Baseline characteristics of CD3^−^CD56^+^ NK cells from the InFoR and EFS control groups. **Table S2.** KIR/HLA genotypes and HLA-ligand combinations in hf-ASD patients, compared with controls from EFS. **Table S3.** IgG serology test of different pathogens in hf-ASD patients and controls from EFS. **Figure S1.** Phenotypic expression of CD56^dim^ and CD56^bright^ NK cell subsets from patients with hf-ASD. **Figure S2.** Expression of supplementary cell-surface markers in NK cells from patients with hf-ASD. **Figure S3.** Functional activity of NK cells from patients with hf-ASD. (DOCX 6113 kb)

## Abbreviations

ASD: Autism spectrum disorders
CMV: Cytomegalovirus
Ctl: Control
DSM-5: Diagnostic and Statistical Manual of Mental Disorders, Fifth Edition
EFS: French National Blood Service
hf-ASD: High-functioning ASD
IFN-γ: Interferon-gamma
IL: Interleukin
IQ: Intelligence quotient
mAb: Monoclonal antibody
NK: Natural killer
PBMCs: Peripheral blood mononuclear cells
TNF-α: Tumor necrosis factor-alpha
