# Supplementary material for: Persistence of dysfunctional natural killer cells in adults with high-functioning autism spectrum disorders: stigma/consequence of unresolved early infectious events?
Source: Mol Autism. 2019 May 15;10:22. doi: 10.1186/s13229-019-0269-1 (PMC6521549; doi:10.1186/s13229-019-0269-1)
Supplement: Supplementary file 1 — Table S1. Baseline characteristics of CD3−CD56+ NK cells from the InFoR and EFS control groups. Table S2. KIR/HLA genotypes and HLA-ligand combinations in hf-ASD patients, compared with controls from EFS. Table S3. IgG serology test of different pathogens in hf-ASD patients and controls from EFS. Figure S1. Phenotypic expression of CD56dim and CD56bright NK cell subsets from patients with hf-ASD. Figure S2. Expression of supplementary cell-surface markers in NK cells from patients with hf-ASD. Figure S3. Functional activity of NK cells from patients with hf-ASD. (DOCX 6113 kb) [file 13229_2019_269_MOESM1_ESM.docx]

**Persistent natural killer cells dysfunctions in adults with high functioning Autism Spectrum Disorders: consequence of unresolved early infectious events?**

**Supplemental Method**

***Detection of Pathogen Stigmata by IgG Elisa Kit in Plasma***

Plasma from 28 hf-ASD individuals and healthy controls from the French National Blood Service (EFS) were assessed for detection by Elisa kit of IgG against Cytomegalovirus (CMV) from NeoBiotech; *Salmonella, Mycoplasma pneumoniae, Bordetella pertussis, Brucella, Borrelia*, Tick-born encephalitis virus, Herpes simplex virus type II, Varicella zoster virus, Rubella virus, Measles virus, Mumps virus, and Hepatitis E virus from Abnova; Adenovirus, Parvovirus B19, and EBV-VCA from Origen; Influenza A virus, Influenza B virus, Parainfluenza virus, and Rous sarcoma virus (RSV) from Fitzge; Hepatitis A virus, Hepatitis D virus, and Hepatitis G virus from Cusabio. Elisa kits were used according to the manufacturer instructions.

**Table S1.** Baseline characteristics of CD3^-^CD56^+^ NK cells from the InFoR and EFS control groups.

|  | InFoR-Ctl | EFS-Ctl | P |
| --- | --- | --- | --- |
| Number | 25 | 35 |  |
| % CD3^-^CD56^+^ | 5.5 ± 3.4 | 9.4 ± 4.2 | 0.0004 |
| % HLA-DR^+^ NK cells | 9.4 ± 3.8 | 4.0 ± 2.9 | 0.0002 |
| % NKp30^+^ NK cells | 79.1 ± 17.4 | 88.5 ± 6.2 | ns |
| % NKp46^+^ NK cells | 52.2 ± 28.0 | 84.8 ± 13.1 | <0.0001 |
| % KIR2DL1^+^ NK cells | 23.6 ± 12.9 | 17.8 ± 6.7 | ns |
| % KIR2DL2/DL3^+^ NK cells | 19.7 ± 8.9 | 29.8 ± 9.9 | 0.0113 |
| % KIR3DL1^+^ NK cells | 17.9 ± 10.4 | 21.2 ± 20.3 | ns |
| % NKG2A^+^ NK cells | 58.6 ± 8.7 | 51.8 ± 10.3 | ns |
| % NKG2C^+^ NK cells | 26.8 ± 18.7 | 5.7 ± 4.2 | <0.0001 |
| % NKG2D^+^ NK cells | 72.4 ± 17.8 | 90.1 ± 9.5 | 0.003 |
| % CD57^+^ NK cells | 31.7 ± 11.2 | 39.7 ± 5.8 | ns |

Data are done in mean ± standard deviation

P values are obtained with a Mann-Whitney test

**Table S2.** KIR/HLA genotypes and HLA-ligand combinations in hf-ASD patients, compared with controls from EFS

|  | EFS-Ctl (n=20) | | hf-ASD (n=35) | | P* |
| --- | --- | --- | --- | --- | --- |
|  | n | % | n | % |  |
| HLA genotype | | | | | |
| C1 | 14 | 0.7 | 28 | 0.8 | ns |
| C2 | 16 | 0.8 | 21 | 0.6 | ns |
| Bw4 | 16 | 0.8 | 31 | 0.9 | ns |
| KIR genotype | | | | | |
| A/A | 6 | 30 | 5 | 14 | ns |
| B/x | 14 | 70 | 30 | 86 | ns |
| Inhibitory KIR | | | | | |
| 2DL1 | 20 | 100 | 34 | 97 | ns |
| 2DL2 | 10 | 50 | 20 | 57 | ns |
| 2DL3 | 18 | 95 | 32 | 91 | ns |
| 2DL5 | 11 | 55 | 23 | 66 | ns |
| 3DL1 | 20 | 100 | 33 | 94 | ns |
| 3DL2 | 20 | 100 | 35 | 100 | ns |
| 3DL3 | 20 | 100 | 33 | 94 | ns |
| Activating KIR | | | | | |
| 2DL4 | 20 | 100 | 35 | 100 | ns |
| 2DS1 | 8 | 40 | 12 | 34 | ns |
| 2DS2 | 10 | 50 | 28 | 80 | 0.033 |
| 2DS3 | 6 | 30 | 22 | 57 | 0.026 |
| 2DS4 | 20 | 100 | 29 | 83 | ns |
| 2DS5 | 6 | 30 | 11 | 31 | ns |
| 3DS1 | 8 | 40 | 15 | 42 | ns |
| KIR-HLA associations | | | | | |
| C2^+^ in 2DL1^+^ | 12/20 | 60 | 20/34 | 59 | ns |
| C2^+^ in 2DS1^+^ | 6/8 | 75 | 9/12 | 75 | ns |
| C1^+^ in 2DL2/DL3^+^ | 14/20 | 70 | 20/35 | 58 | ns |
| Bw4^+^ in 3DL1^+^ | 16/20 | 80 | 32/33 | 97 | ns |

*P: Fisher exact test; ns: non significant

EFS-Ctl: Healthy controls from the French National Blood Service; hf-ASD:

patients with high functioning ASD

**Table S3.** IgG serology test of different pathogens in hf-ASD patients, and controls from EFS

| Pathogens | EFS-Ctl  (N=28) | hf-ASD  (N=28) | P* | P adjusted |
| --- | --- | --- | --- | --- |
| Cytomegalovirus (CMV) | 16 (57.1%) | 9 (32.1) | 0.11 | ns |
| Influenza A | 27 (96.4%) | 27 (96.4%) | 1 | ns |
| Influenza B | 27 (96.4%) | 27 (96.4%) | 1 | ns |
| Para Influenza | 27 (96.4%) | 27 (96.4%) | 1 | ns |
| Rubella virus | 25 (89.3%) | 24 (85.7%) | 1 | ns |
| Varicella zoaster virus | 26 (92.8%) | 26 (92.8%) | 1 | ns |
| Measles virus | 28 (100%) | 28 (100%) | 1 | ns |
| Mumps virus | 26 (92.8%) | 26 (92.8%) | 1 | ns |
| Rous Sarcoma virus (RSV) | 27 (96.4%) | 28 (100%) | 1 | ns |
| Parvovirus B19 | 15 (53.6%) | 17 (60.7%) | 0.79 | ns |
| Adenovirus | 19 (67.8%) | 22 (78.6%) | 0.55 | ns |
| Hepatitis A virus | 12 (42.8%) | 3 (10.7%) | **0.014** | ns |
| Hepatitis C virus | 0 (0%) | 0 (0%) | ns | ns |
| Hepatitis D virus | 0 (0%) | 1 (3.6%) | ns | ns |
| Hepatitis E virus | 1 (3.6%) | 1 (3.6%) | 1 | ns |
| Hepatitis G virus | 0 (0%) | 2 (8.3) | 0.22 | ns |
| Herpes simplex virus type 2 | 21 (75.0%) | 11 (39.3%) | **0.014** | ns |
| Epstein-Barr virus (EBV) | 27 (96.4%) | 22 (78.6%) | 0.11 | ns |
| Tick-born encephalitis virus | 0 (0%) | 0 (0%) | ns | ns |
| *Borrelia* | 1 (3.6%) | 1 (3.6%) | 1 | ns |
| *Brucella* | 4 (14.3%) | 12 (42.9%) | **0.036** | ns |
| *Bordetella* | 14 (50.0%) | 14 (50.0%) | 1 | ns |
| *Mycoplasma* | 22 (78.6%) | 24 (85.7%) | 0.73 | ns |
| *Salmonella* | 1 (3.6%) | 0 (0%) | ns | ns |

* Fisher’s exact test between ASD and healthy controls. ns: non-significant

EFS-Ctl: Healthy controls from the French National Blood Service; hf-ASD: Patients with high

functioning ASD


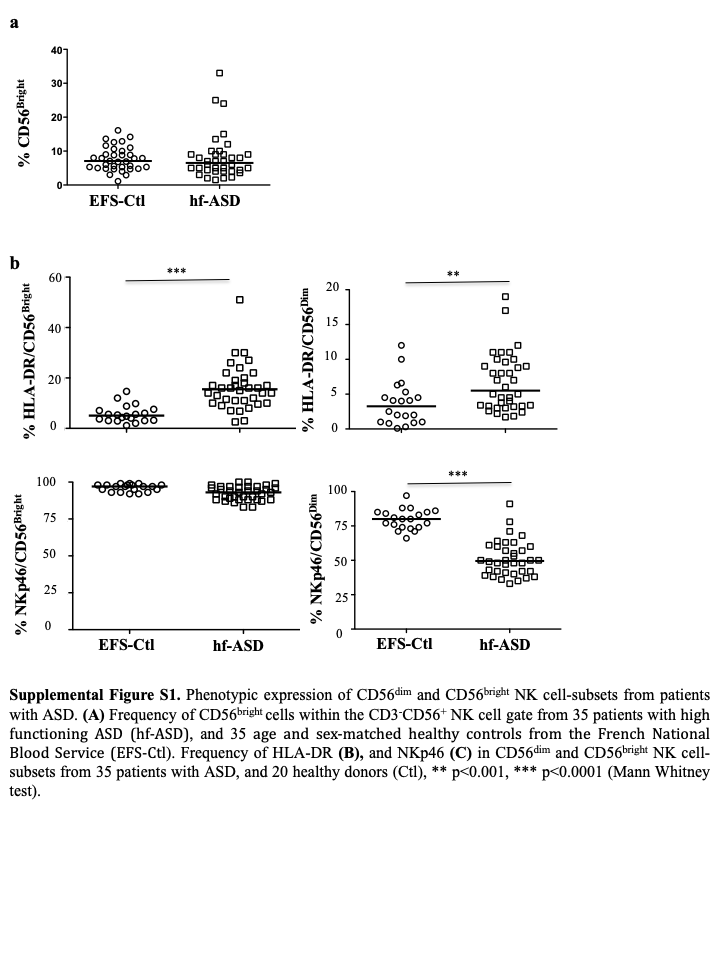


**Fig. S1.** Phenotypic expression of CD56^dim^ and CD56^bright^ NK cell-subsets from patients with hf-ASD. **a** Frequency of CD56^bright^ cells within the CD3^-^CD56^+^ NK cell gate from 35 patients with high functioning ASD (hf-ASD), and 35 age and sex-matched healthy controls from the French National Blood Service (EFS-Ctl). **b** Frequency of HLA-DR and NKp46 in CD56^dim^ and CD56^bright^ NK cell-subsets from 35 patients with ASD, and 20 healthy donors (Ctl). ** p<0.001, *** p<0.0001 (Mann Whitney test).

**
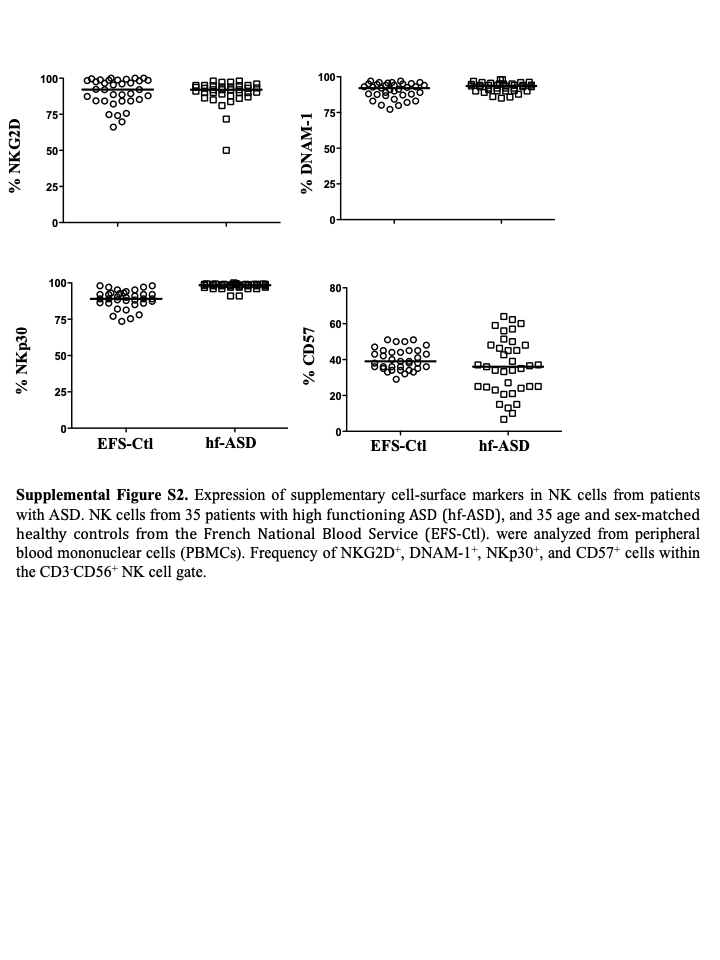
**

**Fig. S2.** Expression of supplementary cell-surface markers in NK cells from patients with hf-ASD. NK cells from 35 patients with high functioning ASD (hf-ASD), and 35 age and sex-matched healthy controls from the French National Blood Service (EFS-Ctl) were analyzed from peripheral blood mononuclear cells (PBMCs). Frequency of NKG2D^+^, DNAM-1^+^, NKp30^+^, and CD57^+^ cells within the CD3^-^CD56^+^ NK cell gate.


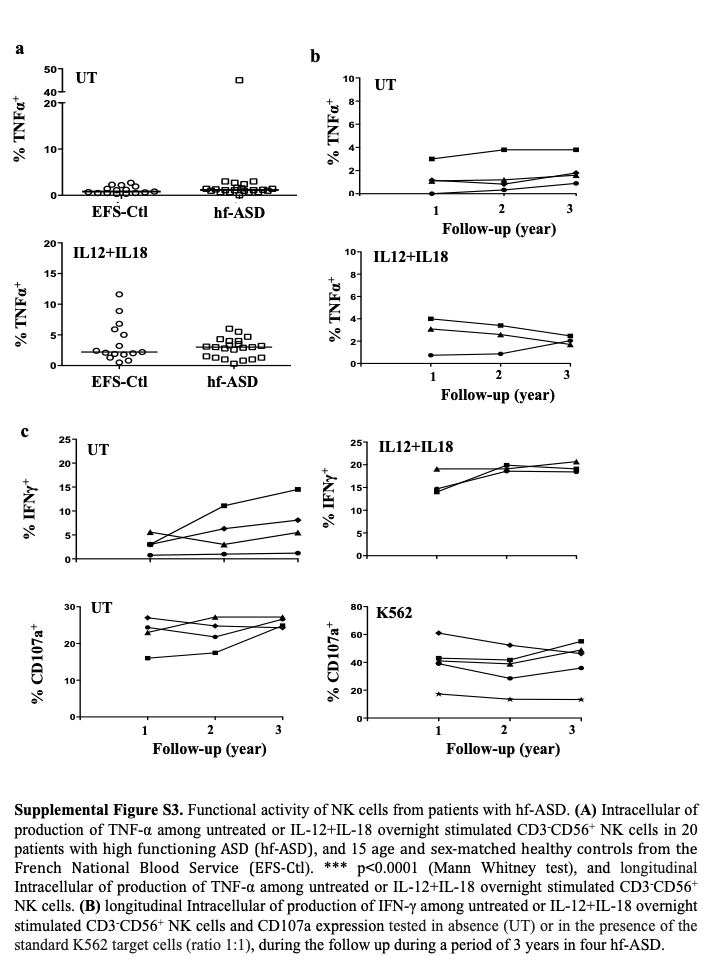


**Fig. S3.** Functional activity of NK cells from patients with hf-ASD. **a** Intracellular production of TNF-α among untreated (UT) or IL-12+IL-18 overnight stimulated CD3^-^CD56^+^ NK cells in 20 patients with high functioning ASD (hf-ASD), and 15 age and sex-matched healthy controls from the French National Blood Service (EFS-Ctl). **b** Longitudinal intracellular production of TNF-α among untreated or IL-12+IL-18 overnight stimulated CD3^-^CD56^+^ NK cells from 4 hf-ASD patients. **c** Longitudinal intracellular production of IFN-γ among untreated (UT) or IL-12+IL-18 overnight stimulated CD3^-^CD56^+^ NK cells and CD107a expression tested in absence (UT) or in the presence of K562 target cells (ratio 1:1), during the follow up during a period of 3 years in four hf-ASD.
